# Impact of COVID-19 Lockdown on the Incidence of Common Pregnancy Complications—Is the Diagnosis of FGR Made Too Generously?

**DOI:** 10.3390/children12081085

**Published:** 2025-08-19

**Authors:** Maximilian Rauh, Silvia Suttner, Claudia Bartl, Marco Weigl, Sven Wellmann, Maurice Kappelmeyer, Börge Schmidt, Maria Emilia Solano, Angela Köninger

**Affiliations:** 1 Department of Obstetrics and Gynecology, Hospital St. Hedwig of the Order of St. John, University of Regensburg, Steinmetzstrasse 1-3, 93049 Regensburg, Germanymaria-emilia.solano@ukr.de (M.E.S.); angela.koeninger@ukr.de (A.K.); 2 Department of Neonatology, Hospital St. Hedwig of the Order of St. John, University of Regensburg, Steinmetzstrasse 1-3, 93049 Regensburg, Germany; sven.wellmann@ukr.de; 3 Laboratory of Translational Perinatology, University of Regensburg, Am Biopark 9, 93053 Regensburg, Germany; 4 Institute of Medical Informatics, Biometry and Epidemiology (IMIBE), University Hospital Essen, University of Duisburg-Essen, Zweigertstrasse 37, 45135 Essen, Germany

**Keywords:** lockdown, COVID-19, pregnancy complications, fetal growth restriction (FGR), pre-pandemic, post-pandemic

## Abstract

**Purpose:** In 2020, a lockdown due to COVID-19 was ordered by the German government, resulting in population-wide restrictions. In this retrospective study, we question the extent to which health policy restrictions have influenced medical diagnoses. **Methods:** The incidence rates of relevant pregnancy complications during all trimesters of pregnancy were evaluated for a 6-month pre-pandemic period (April–September 2019), in comparison to the same period during the lockdown in 2020. Mothers and newborns who presented at the University Hospital St. Hedwig, Regensburg, Germany, were included in the study. The incidence rates of preeclampsia and suspected FGR (fetal growth retardation), as relevant obstetric diseases, were further compared with those in a post-pandemic period (April–September 2023). **Results:** A total of 5137 newborns were included, with 1709 born during the 6-month pre-pandemic period, 1806 during the 6-month lockdown period and 1687 during the 6-month post-pandemic period. During the pandemic period, significantly fewer patients were hospitalized due to hyperemesis gravidarum (1.8% vs. 0.9%, *p* = 0.04). No differences were observed concerning the incidence of miscarriages before and after 14 weeks of gestation (WG), preterm deliveries (<37 WG), gestational age at preterm birth and birth weight. Likewise, within the group of preterm-born babies, no difference was observed in preeclampsia among the two periods. However, in the pandemic period, the frequency of preterm-born babies with suspected FGR was significantly lower than in the pre-pandemic period (1.5% and 0.6%, *p* = 0.01). Regarding this point, we analyzed data of all newborns in a comparative post-pandemic period in 2023 (n = 1687). This group presented a significantly increased FGR incidence compared to that during the pandemic, therefore returning to the pre-pandemic level (1.5% and 1.4%, *p* = 0.145). **Conclusions:** During the COVID-19 pandemic, there were significantly fewer deliveries with suspected FGR. After all specific restrictions on elective and outpatient services were lifted, the incidence of suspected FGR returned to its initial level, suggesting that the diagnosis—as a solely machine-dependent and not symptom-based diagnosis—was partly exaggerated in both the pre-and post-pandemic periods.

## 1. Introduction

In December of 2019, an outbreak of the disease caused by coronavirus SARS-CoV-2 (COVID-19) was registered in the Chinese city of Wuhan. Thereafter, the disease spread rapidly worldwide and became a pandemic.

The first case of COVID-19 in Germany was confirmed on the 27 January 2020 [1]. In mid-March of 2020, the German government ordered a national lockdown, including the closure of schools, non-essential shops and businesses, and a ban on attending nursery homes and hospitals. Later on, curfews were imposed and physical contact with more than one person from outside one’s household was prohibited. The healthcare systems confronted a challenging situation. To meet the needs of the emerging number of COVID-19 patients, restrictions were placed on elective and outpatient services.

As maternity care is an essential service, it was continuously offered to pregnant patients throughout the lockdown phase. Adjustments were made where possible, including reducing the number of in-person visits, restricting accompanying visitors, and rescheduling or even postponing maternal and fetal assessment appointments [2]. It is known, from other European countries, that the number of examinations requiring direct contact with a doctor decreased [3,4,5].

To date, there is no doubt that the SARS-CoV-2 infection affects maternal and child health directly [6]. However, the indirect effects of the pandemic on pregnancy outcomes (e.g., due to the national lockdown) are less clear. Fear of seeking medical healthcare as well as the reduced provision of routine antenatal visits have been suggested as probable causes of adverse perinatal outcomes [7]. Several reports have indicated that the rates of stillbirths and preterm births might have changed significantly during the lockdown and pandemic phases [8,9,10,11,12,13,14,15,16,17,18,19,20]. Likewise, the named changes in the maternity care services, as well as maternal stress and anxiety during the lockdown may have also affected the progress of pregnancy.

Hence, the goal of the present study was to investigate whether the strict lockdown at the beginning of the SARS-CoV-2 pandemic in Germany influenced the incidence of selected pregnancy complications of high frequency in our tertiary maternity care hospital.

## 2. Material and Methods

In this retrospective observational study, selected common pregnancy complications of patients treated at the University Hospital St. Hedwig, Regensburg, Germany, were compared during 3 periods: from 1 April 2020 to 30 September 2020 (the time during the strict lockdown and shortly after; the so-called “lockdown” or “pandemic” period), from 1 April 2019 to 30 September 2019 (the so-called “pre-pandemic period”) and from 1 April 2023 to 30 September 2023 (the so-called “post-pandemic period”).

Regarding early pregnancy, the number of hospitalized patients suffering from hyperemesis gravidarum and miscarriages before 14 + 0 weeks of gestation (WG) was analyzed. Hyperemesis gravidarum was defined as excessive and persistent vomiting in the first trimester of pregnancy, which was assessed using the PUQE score [21]. Miscarriages before and after 14 + 0 weeks of gestation (WG) were analyzed separately. Preterm birth was defined as a gestational age between 22 + 0 and 36 + 6 WG [22]. The provision of maximum care is standard for neonates born after 24 + 0 WG. Medical care for extremely premature births from 22 + 0 WG is dependent on several factors, such as estimated fetal weight or presence of a maternal infection. Parental decision on palliative or maximum neonatal care before 24 + 0 WG is made following discussion with an experienced neonatologist.

We additionally analyzed the incidence of preeclampsia and FGR (fetal growth retardation), as common complications in pregnancy. Preeclampsia was defined as chronic or gestational hypertension with at least one new organ manifestation during pregnancy that could not be attributed to any other cause [23]. Patients with HELLP (acronym from: hemolysis, elevated liver enzymes and reduced platelet count) syndrome were also included in this group [24].

Furthermore, we used the DELPHI criteria to define FGR and differentiate it from small for gestational age (SGA) [25,26]. We took the prenatal diagnosis into account, and not the final weight of the newborn.

The sonographic examination was carried out using a high-resolution convex transducer (3.5 mHz). Voluson S8, E8 and E10 ultrasound machines were used (GE Healthcare GmbH, 80807 München, Germany). The examinations were carried out in accordance with everyday clinical practice by experienced sonographers. The fetal parameters were documented using the ViewPoint program (ViewPoint™, GE Healthcare GmbH, 42655 Solingen). Weight estimation is routinely performed using Hadlock’s estimation formula, which is based on measurements of biparietal diameter (BPD), head circumference (HC), abdominal circumference (AC) and femur length (FL), calculated as log10 G = 1.3596 − 0.00386 × AC × FL + 0.0064 × HC + 0.00061 BPD × AC + 0.0424 × AC + 0.174 × FL, which is referred to as the “Hadlock I” [27].

All pregnant women who were treated in the respective epoch due to the named diagnosis were included. For this purpose, ICD-10 codes were surveyed retrospectively. In addition, a comparison was made with the analogue documentation that had been carried out. Exclusion criteria such as age, body mass index (BMI) or ethnicity were not applied. All live births during the relevant period were included in the study—both singleton and multiple pregnancies.

All parameters and measured values were collected retrospectively and finally drawn from the digital archive (SAP, Viewpoint).

**Figure 1:**
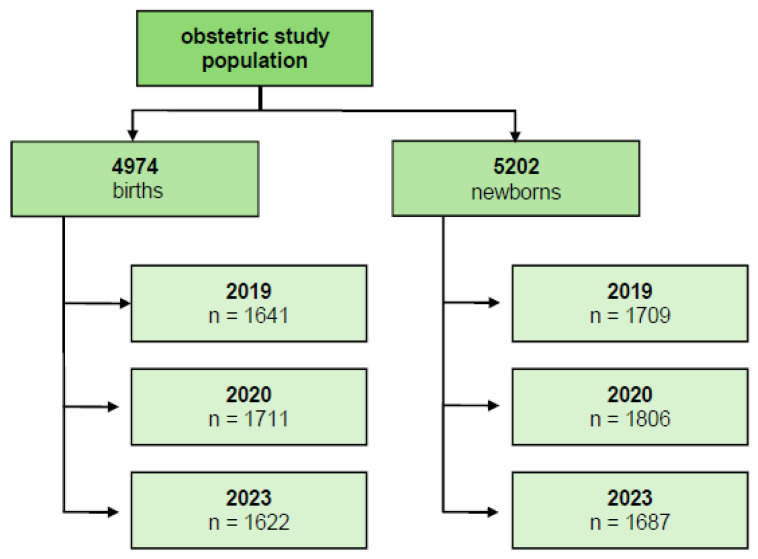
Included births and newborns.

**Figure 2:**
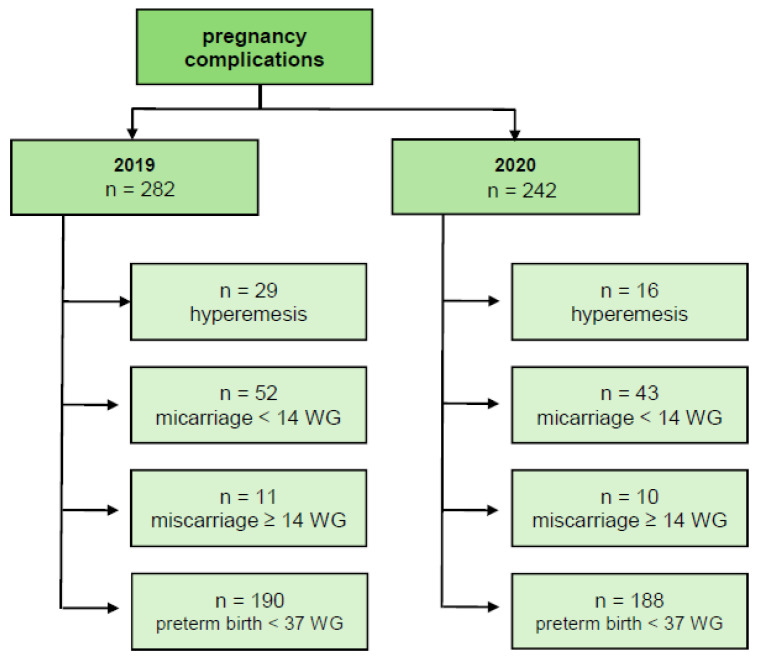
Included patients with pregnancy complications.

The reference value for pregnancy complications was the number of births in the respective period. The reference value for preterm births was the number of newborns in the respective period (Figure 1 and Figure 2).

After initial analysis, a further subgroup analysis was performed with regard to the incidence of FGR and preeclampsia in the post-pandemic period.

Group comparisons by mean were performed using the *t*-test. The Chi-Square test was used for categorical data when the expected cell counts were higher than 5; otherwise, Fisher’s exact test was used. To calculate 95% confidence intervals for odds ratios, the Wald test was carried out. The analysis was performed using R version 4.4.1. A *p*-value < 0.05 was considered statistically significant.

The study was approved by the ethics committee of the University of Regensburg (No. 24-4004-104).

## 3. Results

A total of 5202 newborns were included, with 1709 born during the 6-month pre-pandemic period, 1806 during the 6-month lockdown (i.e., pandemic) period and 1687 during the analyzed 6-month post-pandemic period. In the subgroup of preterm births, gestational age was between 23 + 5 WG and 36 + 6 WG. We first evaluated whether maternal age differed within the analyzed pregnancy complication categories among the pre-pandemic and lockdown study populations. No differences in maternal age between all categories (Table 1) were observed and, therefore, we did not adjust for this variable as a confounder.

During the pandemic period, there was a significantly (*p* = 0.04) lower frequency of admissions to the hospital due to hyperemesis compared to the pre-pandemic period: 0.9% (16/1711) vs. 1.8% (29/1641) (Table 2). In particular, at the beginning of the national mandated curfews (from 1 April until 12 May 2020)—due to the strict policy limiting contact to a maximum of one person outside of a household—there were no admissions to the hospital due to hyperemesis.

Compared to the pre-pandemic period, there was a trend towards a reduced rate of miscarriages both before and after 14 WG in the pandemic period; however, statistical significance was not reached. There were 2.6% (43/1711) miscarriages with curettage (<14 WG) in the lockdown vs. 3.2% (52/1641) in the pre-pandemic period (*p* = 0.25), and 0.5% (10/1711) miscarriages (≥14 WG) in the lockdown vs. 0.7% (11/1641) in the pre-pandemic period (*p* = 0.75) (Table 2). As shown in Table 1, maternal age differed significantly between the subgroup with miscarriages > 14 + 0 WG, tending toward older women in the pre-pandemic period. This may be associated with the higher rate of known or visible fetal anomalies in the pre-pandemic period (6 anomalies among 11 miscarriages) compared to that in the lockdown period (3 anomalies among 9 miscarriages). Due to the fact that chromosomal analysis was not performed in all miscarriage cases and considering the small number of cases, we did not further discriminate our analysis between spontaneous labor of a viable fetus and intrauterine fetal demise.

The rate of preterm birth (<37 + 0 WG) showed no statistical difference between the two periods. In the pre-pandemic period, 13.1% (224/1709) of the children were born preterm, whereas 12.0% (217/1806) were preterm in the lockdown period (*p* = 0.33) (Table 2). Within the preterm birth group, we compared the birth weight and gestational age between the children born in the two periods. There was no significant difference between the gestational age at delivery. The mean gestational age of the preterm-born group in the pre-pandemic period was 237 days (SD 23.0), while that in the lockdown period was 233 days (ISD 22.2) (*p* = 0.10). Similarly, there was no significant difference between the birth weight of preterms born alive: the mean birth weight was 2163 g (SD 637.2) in the pre-pandemic period, while that during the lockdown period was 2240 g (SD 716.7) (*p* = 0.23). An in-depth comparison of the subsequent weight categories revealed no significant differences between the two periods (Table 3). Due to their low frequency, we included all babies with birth weight ≤ 1000 g in one group. Among them, only 3 and 2 neonates with birth weight ≤ 500 g were delivered in the pre-pandemic and pandemic periods, respectively.

A subgroup analysis of preterm-delivered babies (224 in the pre-pandemic period, 217 in the pandemic period) was performed according to common pregnancy complications. Interestingly, the number of preterm deliveries with suspected FGR decreased by more than 50% in the lockdown period: the rate of preterm birth with FGR was 0.5% (9/1806) during the lockdown vs. 1.5% (23/1709) in the pre-pandemic period (*p* = 0.01). Furthermore, the rate of preterm birth with preeclampsia was 1.5% (27/1806) in the lockdown period vs. 1.3% (24/1709) in the pre-pandemic period (*p* = 0.82) (Table 2).

As the incidence of FGR cases in preterm deliveries differed significantly between the pre-pandemic and pandemic periods, we performed an additional subgroup analysis of preterm FGR cases in 2023. In this post-pandemic period, the rate was 1.5% (26/1687), compared to the rate of 0.5% (9/1806) observed during the pandemic period (*p* = 0.005). This reflects a return to the pre-pandemic incidence of FGR in preterms—there was no significant difference between the FGR rates in 2019 (1.3%, 23/1709) and 2023 (1.5%, 26/1687) (*p* = 0.54) (Table 4).

Interestingly, there was no significant change in the proportional incidence of early-onset FGR (<32 WG) and late-onset FGR (>32 WG) between the three epochs (Table 5). And also the rate of stillbirths did not change significantly in the corresponding periods (Table 6). 

In contrast, the incidence of preeclampsia within the preterm-born cohort did not differ significantly between the pre-pandemic, pandemic and post-pandemic periods (Table 4).

The mode of delivery in the subgroup of preterm-born FGR fetuses did not differ significantly between the pre-pandemic, pandemic and post-pandemic periods (*p* = 0.66): in the pre-pandemic period, 87% (20/23) of the women had a cesarean section and 13% (3/23) gave birth vaginally. In the pandemic period, these percentages were 77.8% (7/9) versus 22.2% (2/9) and, in the post-pandemic period, these percentages were 76.9% (20/26) versus 23.1% (6/26) (Table 7).

The birth weight of preterm infants with FGR differed significantly between the analyzed periods (*p* = 0.019). Comparing the prenatal diagnosis with the final postnatal diagnosis, 78.2% (18/23) of the newborns classified as FGR prenatally had a birth weight below the 10th percentile in the pre-pandemic period. In the lockdown and post-pandemic periods, 100% (9/9) and 53.8% (14/26) were born with a weight below the 10th percentile, respectively (Table 6).

The indication for preterm delivery changed significantly between the pre-pandemic, lockdown and post-pandemic periods (*p* = 0.045). In the pre-pandemic period, 60.8% (14/23) of the FGR fetuses were delivered due to FGR. In the remaining cases FGR was a suspected additional diagnosis but was not the reason for delivery: 21.7% (5/23) were delivered due to preeclampsia and 17.5% (4/23) due to premature rupture of the membranes (PROM) or labor. In the pandemic period, 66.7% (6/9) were delivered due to FGR, 22.2% (2/9) due to preeclampsia and 11.1% (1/9) due to premature labor. In the post-pandemic period, 42.3% (11/26) were born because of FGR, 15.4% (4/26) due to preeclampsia, 38.5% (10/26) due to PROM or premature contractions and 3.8% (1/26) due to uterine rupture after previous cesarean section (Table 6).

## 4. Discussion

In this study, the incidence of common pregnancy complications in a single center before, during and after the lockdown phase of the COVID-19 pandemic was compared. In general, the incidence of all analyzed pregnancy complications showed a trend of decreasing during the pandemic period when compared to the pre-pandemic period. While we found no difference between miscarriage rates, there was a significant reduction in inpatient care due to hyperemesis. The rate of preterm-born babies (<37 WG) did not significantly differ, nor gestational age or birth weight in preterm-born babies. The number of early deliveries due to preeclampsia, preterm contractions or premature rupture of membranes did not differ between the studied periods. However, the overall incidence of preterm-born babies with suspected FGR was significantly lower during the pandemic period. The incidence of preterm birth with FGR and preeclampsia was therefore further analyzed in a post-pandemic period. Interestingly, the incidence of suspected FGR in the post-pandemic period—namely, after all restrictions on elective and outpatient services had been lifted—returned to the pre-pandemic level.

There are only limited data on the direct and indirect effects of the SARS-CoV-2 pandemic on first- and early second-trimester pregnancy complications and outcomes.

A study performed in Turkey including 1260 women showed a significantly higher rate of miscarriages < 12 WG during the pandemic than pre-pandemic (11.8% versus 9.2%, *p* = 0.0001), although positivity for SARS-CoV-2 did not have a significant effect on the miscarriage rate (*p* = 0.81) [28]. One case–control study from Italy including 225 women (100 with spontaneous abortion and 125 with ongoing pregnancy) found no difference in the cumulative incidence of COVID-19 between women with miscarriage and those with ongoing pregnancy (11% versus 9.6%, *p* = 0.73) [15]. This is in line with the findings of our study: miscarriage rates before and after 14 + 0 WG did not differ between the compared epochs in 2019 (pre-pandemic) and 2020 (pandemic period).

However, we observed a significant reduction in admissions to the hospital due to hyperemesis in the 6-month study period during 2020 compared to 2019. We hypothesize that the fear of harm to the child due to hyperemesis is not a main concern for pregnant women and, therefore, the threshold for seeking medical help regarding their own wellbeing declined in the lockdown phase. Likewise, decreased admission rates for complaints associated with pregnancy have been reported in other studies. In a Israeli study, the authors observed a major decline in all aspects of routine obstetric activities during the pandemic period. Visits to obstetric triage, gynecologic triage, high-risk clinic and ultrasound units decreased by 36.4%, 34.7%, 32.8% and 18.1%, respectively, compared to the same period in the previous (pre-pandemic) year [29]. In a Polish cross-sectional study, Czuba et al. analyzed datasets of 237,396 women participating in a prenatal screening program (126,552 pre-pandemic and 110,844 pandemic). They demonstrated that COVID-19-related restrictions affected prenatal diagnostics: the number of women attending first-trimester prenatal visits decreased, while tests not requiring direct contact (e.g., triple test) became more frequent [3]. In a retrospective single center study in Israel including data from 1556 deliveries during 2020 (pandemic) and 4564 deliveries between 2017 and 2019, the authors observed a decline in attendance to the obstetrical emergency room (both by daily count and per woman), in comparison to combined matched periods in 2017, 2018 and 2019 [4]. In an Italian study, the authors examined the effects of COVID-19 on gynecological emergency admissions. They included 9291 data (5644 pre-pandemic, 3647 pandemic) and observed that the lockdown reduced admissions and therefore negatively influenced women’s health [5].

As far as the rate of premature births is concerned, initial international studies have reported declines, particularly in terms of very low birth weight. In a retrospective Irish analysis of 30,705 births between 2001 and 2020, a rate of 2.17 infants with very low birth weight per 1000 live births was observed during the pandemic compared to 8.18, representing a reduction of 73% (*p* = 0.022) [20]. In a nationwide register-based study focused on 31,180 live-born singletons in Denmark, a significantly lower rate of extremely premature infants (≤27 + 6 WG) was found during the lockdown period compared with the corresponding period in previous years; in particular, there were 0.19 extremely premature births per 1000 births during the lockdown period compared with an average of 2.19 in previous years [11]. Been et al. included data from 1,599,547 singleton neonates who had undergone neonatal blood spot screening in the Netherlands between 2010 and 2020, including 56,720 births during the lockdown period. The reduction was consistent across various degrees of prematurity [8]. In a Israeli study, the authors compared the outcomes of 34,022 singleton pregnancies (2594 pandemic; 2742 pre-pandemic in 2019; 28,686 pre-pandemic in matched periods between 2011 and 2019). They observed a significantly lower preterm birth rate < 34 + 0 WG in the pandemic period (12 per 1000 births versus 27 in 2019 versus 21 in matched periods, *p* = 0.004) [19]. In an African study in Botswana, 68,448 women who delivered a singleton in 2017–2020 between 1 January and 20 July were included. A 9% relative reduction in preterm births was observed in the pandemic period [9].

We could not confirm the above results in our study. However, our result of a constant premature birth rate (13.1% pre-pandemic versus 12% pandemic, *p* = 0.33) is consistent with the results of other studies. In an American study, the authors compared singleton preterm birth rates across racial and ethnic groups during the COVID-19 pandemic in California. They included 713,567 data (132,853 during the pandemic; 580,714 from 2016 to 2019). The overall rate of births < 37 WG was unchanged [18]. In another American cohort study, data of 8914 deliveries in Philadelphia (5907 pre-pandemic, 3007 pandemic) were included. There were no significant interactions between ethnicity or period with spontaneous preterm births (57 versus 47 per 1000 live births, *p* = 0.09) [14]. In the abovementioned Israeli study conducted by Mor et al., the rate of premature delivery did not differ significantly between all groups (<28, <34, <37 weeks’ gestation) [4]. In a German study, the authors also observed limited effects of the lockdowns on preterm birth rates. They included 361,737 preterm births (35,333 during the lockdown in 2020, 326,404 in corresponding periods between 2010 and 2019). Singleton preterm births < 32 + 0 WG were significantly decreased during the first lockdown period (0.9% versus 0.72%, *p* = 0.04), but not in the second lockdown. There was no difference in preterm births < 28 + 0 WG. For twin births, there was no significant difference in both lockdown periods [30].

Preterm birth with preeclampsia showed a subtle increase in incidence during the pandemic period. It has been reported that COVID-19 infection during pregnancy is strongly associated with preeclampsia [31]. However, neither the overall incidence of preeclampsia nor the rate of deliveries due to preeclampsia differed in our study cohort.

Finally, the decrease in suspected FGR in our study was unexpected, as it has been demonstrated that SARS-CoV-2 infection during pregnancy is associated with a higher incidence of low placental weight and an increased birth weight/placental weight ratio, regardless of which trimester the infection occurred [32]. Comparing the incidence of FGR in our study (0.5–1.5%), it is slightly lower than described by Świercz et al. in a Polish study 2024. The authors performed a follow-up on 1212 patients who underwent first-trimester prenatal screening (2018–2019). FGR occurred in 2.2% of cases (34.6% before and 65.4% after 32 weeks’ gestation) [33].

The reduced access to prenatal services in combination with the fear of seeking medical care seems to have led to a more reasonable use of resources avoiding the diagnostic overcall of other time periods. In the end this resulted in less iatrogenic preterm birth with diagnosis of FGR. This hypothesis is supported by the fact that the post-pandemic incidence of FGR was similar to the pre-pandemic incidence in our study. FGR is a risk factor for stillbirth, and several international reports have demonstrated an increased rate of stillbirth during the pandemic period.

In an Italian retrospective data analysis of 16,808 singleton births, the stillbirth rate increased from 1.07 to 3.23 per 1000 births during this period (*p* = 0.0017) [10]. Khalil et al. observed a significant increase in the stillbirth rate when comparing data of 1681 pre-pandemic and 1718 pandemic singleton and multiple births at St. George’s University Hospital in London (9.31 versus 2.38 per 1000 births; *p* = 0.01) [9]. In a prospective observational study in Nepal including 21,763 women, the rate of stillbirths increased significantly from 14 pre-pandemic to 21 per 1000 births during the lockdown period (*p* = 0.0002) [12]. Kumar et al. performed a case–control study including 9771 deliveries (6161 pre-pandemic; 3610 during lockdown) in a tertiary care center in India. They observed a significant increase in the rate of stillbirths during lockdown (37.4 versus 29.9 per birth, *p* = 0.045). They mainly attributed this increase to delays in reaching the hospital and undertaking operative procedures [16]. In another Indian retrospective study across four hospitals in an integrated tertiary care medical college, the authors observed a decrease in hospital admissions of pregnant women during the lockdown period, accompanied by an increase in the stillbirth rate (31.5 versus 22.5 per 1000 births, *p* = 0.02) [17].

However, there are also conflicting results: in an Israeli study, the authors compared the outcomes of 34,022 singleton pregnancies (2594 pandemic, 2742 pre-pandemic in 2019, 28,686 pre-pandemic in matched periods between 2011 and 2019) and found that the stillbirth rates did not differ (8 versus 8 versus 10 per 1000 births, *p* = 0.424) [19]. Additionally, in the abovementioned American study conducted by Hanley et al., there was no difference in the stillbirth rate. They observed a rate of 5.4 stillbirths per 1000 births in the pre-pandemic period versus 5.0 stillbirths per 1000 births in the pandemic period (*p* = 0.88) [14]. This is consistent with our finding that there is no significant change in the stillbirth rate compared to either the birth rate or the newborn rate. Also an analysis of the Bavarian birth cohort—including our cases—indicated no increase in the stillbirth rate during the lockdown period. Stumpfe et al. performed a analysis including 349,245 births from 2010 to 2020. While the rate of stillbirths was significantly higher during the first lockdown when compared to pre-pandemic reference periods (4.04 versus 3.03 per 1000 births, *p* = 0.03), this effect did not persist after adjusting for long-term and seasonal trends. Furthermore, the rate of stillbirths was not significantly different during the second lockdown when compared to the pre-pandemic periods (3.46 versus 2.93 per 1000 births) [34].

Apart from established sonographic fetal weight estimation, Doppler ultrasound and CTG controls the antenatal umbilical coiling index (aUCI) might be useful to further stratify the risk of FGR. In a Vietnamese cross-sectional study on 337 full-term singletons Nguyen et al. demonstrated that fetuses with hypocoiling (n = 40) or hypercoiling (n = 71) of the umbilical cord have a significantly higher risk of adverse pregnancy outcomes (e.g., abnormal fetal heart rate, Apgar scores at 5 min below 7) [35].

Aside from less iatrogenic preterm birth with FGR diagnosis, another hypothesis to explain the decreased incidence of FGR births is that the lockdown and pandemic resulted in behavioral and lifestyle modifications, which may have had an influence on health and wellbeing. Philip et al., in a retrospective analysis of 30,705 births between 2001 and 2020, reported a rate of 2.17 infants with very low birth weight per 1000 live births during the pandemic compared to 8.18 in the pre-pandemic period, representing a reduction of 73% (*p* = 0.022) [20].

## 5. Strength and Limitations

The strength of our study is the subgroup analysis of preterm birth indications in a total of more than 5000 newborns.

Limitations of our study include its retrospective design and being carried out at a single institution. The risks associated with the retrospective design include possible miscoding and lack of precision in the reported diagnoses of medical conditions. Furthermore, we did not adjust for basic characteristics such as parity and obstetric history (e.g., preterm birth history), which might have acted as confounders in the analysis.

Furthermore, we did not assess the percentage of confirmed COVID-19-positive mothers in this study or the total incidence of preeclampsia as well as FGR (term and preterm), and we did not discuss the influence of coronavirus vaccination, which was available in Germany from 26 December 2020 [36].

## 6. Conclusions

In this study on effects of selected common pregnancy complications of the national COVID-19 pandemic lockdown the incidence of pregnancies with suspected FGR was significantly lower during the pandemic than in the pre-pandemic period. After all COVID-19-restrictions had been lifted, the incidence of suspected FGR returned to its pre-pandemic level.

In contrast to all other analyzed pregnancy complications, prenatal FGR diagnosis is solely machine-dependent and does not rely on clinical diagnosis. The results demonstrate how physicians’ diagnoses and their impacts on clinical outcomes were apparently influenced by the health policy restrictions. The results raise the question regarding whether the diagnosis of FGR results in overly generous iatrogenic preterm delivery.

## Figures and Tables

**Table 1:** Maternal age in the study population.

	Pre-Pandemic Period Maternal Age (Years) Mean (SD) Median (IQR) n = Total Number	Pandemic Period Maternal Age (Years) Mean (SD) Median (IQR) n = Total Number	*p*-Value
Whole cohort of patients with pathology	32.0 (5.3) 32.0 (28.0–36.0) n = 282	31.8 (5.7) 31.0 (28.0–35.5) n = 257	0.67
**Complication/Outcome:**			
Hyperemesis	29.9 (5.6) 31.0 (25.0–33.5) n = 29	28.7 (5.8) 27.5 (24.5–34.5) n = 16	0.50
Miscarriage < 14 + 0 WG	34.1 (5.7) 33.5 (30.3–38.0) n = 52	33.1 (5.3) 34.0 (29.0–37.0) n = 43	0.38
Miscarriage ≥ 14 + 0 WG	33.1 (5.1) 32.0 (29.0–37.0) n = 11	28.9 (3.2) 30.0 (26.0–32.0) n = 10	0.04 *
preterm birth (<37 + 0 WG)	31.7 (5.0) 31.0 (28.0–35.0) n = 190	31.9 (5.8) 31.0 (28.5–35.0) n = 188	0.72
**Preterm birth subgroups:**			
Preterm with suspected FGR	31.0 (5.8) 30.5 (27.5–36.0) n = 23	32.9 (4.7) 31.0 (29.5–36.0) n = 9	0.39
Preterm with preeclampsia	30.5 (4.3) 29.0 (27.5–33.5) n = 24	33.4 (5.9) 34.0 (28.0–36.3) n = 27	0.053

Significance was calculated via the *t*-test. * *p* < 0.05 was considered statistically significant. IQR: interquartile range; SD: standard deviation.

**Table 2:** Pregnancy complications and outcomes in the pre-pandemic (April to September 2019) and lockdown (April to September 2020) periods.

Complications/Outcome	Pre-Pandemic Period n (%)	Pandemic Period n (%)	*p*-Value	OR (95% CI)
**Hyperemesis** *(pre-pandemic: births = 1641* *pandemic: births = 1711)*	29 (1.8%)	16 (0.9%)	0.04 *	**1.90 (1.03–3.52)**
**Miscarriage < 14 + 0 WG** *(pre-pandemic: births = 1641* *pandemic: births = 1711)*	52 (3.2%)	43 (2.6%)	0.25	1.27 (0.84–1.91)
**Miscarriage ≥ 14 + 0 WG** *(pre-pandemic: births = 1641* *pandemic: births = 1711)*	11 (0.7%)	10 (0.5%)	0.75	1.14 (0.49–2.71)
**Preterm birth (<37 + 0 WG)** *(pre-pandemic: newborns = 1709 * *pandemic: newborns = 1806)*	224 (13.1%)	217 (12.0%)	0.33	1.10 (0.90–1.34)
Preterm birth subgroups:				
**Preterm with suspected FGR** *(pre-pandemic: newborns = 1709 * *pandemic: newborns = 1806)*	23 (1.5%)	9 (0.5%)	0.01 *	**2.72 (1.26–5.90)**
**Preterm with preeclampsia** *(pre-pandemic: newborns = 1709 * *pandemic: newborns = 1806)*	24 (1.3%)	27 (1.5%)	0.82	0.93 (0.53–1.63)

Significance was calculated via the Chi-Square test. * *p* < 0.05 was considered statistically significant.

**Table 3:** Birth weight categories of preterm-born babies (<37 + 0 WG).

Birth Weight (g)	Pre-Pandemic Period n (%) (Total Number of Preterms 224)	Pandemic Period n (%) (Total Number of Preterms 217)	*p*-Value	OR (95% CI)
<1000	14 (6.3%)	15 (6.9%)	0.93	0.90 (0.42–1.90)
1000–<1500	22 (9.8%)	20 (9.2%)	0.95	1.07 (0.57–2.02)
1500–<2000	42 (18.7%)	29 (13.4%)	0.16	1.50 (0.89–2.50)
2000–2500	68 (30.4%)	67 (30.9%)	0.99	0.97 (0.65–1.46)
>2500	78 (34.8%)	86 (39.6%)	0.34	0.81 (0.55–1.20)

Significance was calculated via the Chi-Square test.

**Table 4:** Comparison of the incidence of FGR and preeclampsia in pre-pandemic, pandemic and post-pandemic periods.

	Pre-Pandemic Period *(Total Number of Newborns = 1709)* n (%)	Pandemic Period *(Total Number of Newborns = 1806)* n (%)	Post-Pandemic Period *(Total Number of Newborns = 1687)* n (%)	*p*-Value
Preterm born babies with FGR	23 (1.3%)	9 (0.5%)	26 (1.5%)	0.005 *
Preterm born babies with preeclampsia	24 (1.4%)	27 (1.5%)	13 (0.8%)	0.145

Significance was calculated via the Chi-Square test. * *p* < 0.05 was considered statistically significant.

**Table 5:** Comparison of the incidence of early- and late-onset FGR in the pre-pandemic, pandemic and post-pandemic periods.

	Pre-Pandemic Period *(n = 23)* *n (%)*	Pandemic Period *(n = 9)* *n (%)*	Post-Pandemic Period *(n = 26)* *n (%)*	*p*-Value
Early-onset	11 (48%)	3 (33.3%)	7 (27.9%)	0.31
Late-onset	12 (52%)	6 (66.7%)	19 (72.1%)	

Significance was calculated via Fisher’s exact test. *p* < 0.05 was considered statistically significant.

**Table 6:** Comparison of the incidence of stillbirths in the pre-pandemic, pandemic and post-pandemic periods.

	Pre-Pandemic Period *n (%)*	Pandemic Period *n (%)*	Post-Pandemic Period *n (%)*	*p*-Value
**Stillbirths** *(pre-pandemic newborns = 1709* *pandemic newborns = 1806* *post-pandemic newborns = 1687)*	8 (0.47%)	9 (0.50%)	8 (0.47%)	0.86

Significance was calculated via Fisher’s exact test. *p* < 0.05 was considered statistically significant.

**Table 7:** Preterm-born infants with suspected FGR.

	Pre-Pandemic Period (n = 23)	Pandemic Period (n = 9)	Post-Pandemic Period (n = 26)	*p*-Value
**Mode of delivery**				
Cesarean section	20 (87%)	7 (77.8%)	20 (76.9%)	0.66
Vaginal birth	3 (13%)	2 (22.2%)	6 (23.1%)	
**Birth weight**				
<10th percentile	18 (78.2%)	9 (100%)	14 (53.8%)	0.019 *
≥10th percentile	5 (21.7%)	0 (0%)	12 (46.2%)	
**Indication for delivery**				
FGR	14 (60.8%)	6 (66.7%)	11 (42.3%)	0.045 *
Preeclampsia	5 (21.7%)	2 (22.2%)	4 (15.4%)	
PPROM/contractions	4 (17.5%)	1 (11.1%)	10 (38.5%)	
Others	0 (0%)	0 (0%)	1 (3.8%)	

Significance was calculated via Fisher’s exact test. * *p* < 0.05 was considered statistically significant.

## Data Availability

The datasets used and/or analyzed during the current study are available from the corresponding author on reasonable request. The data are not publicly available due to ethical and privacy concerns.

## Abbreviations

COVID-19	coronavirus disease 2019
IQR	interquartile range
SARS-CoV-2	severe acute respiratory syndrome coronavirus 2
SD	standard deviation
WG	weeks of gestation
